# Gossip and reputation in everyday life

**DOI:** 10.1098/rstb.2020.0301

**Published:** 2021-11-22

**Authors:** Terence D. Dores Cruz, Isabel Thielmann, Simon Columbus, Catherine Molho, Junhui Wu, Francesca Righetti, Reinout E. de Vries, Antonis Koutsoumpis, Paul A. M. van Lange, Bianca Beersma, Daniel Balliet

**Affiliations:** ^1^ Department of Experimental and Applied Psychology, Institute for Brain and Behaviour Amsterdam (IBBA), Vrije Universiteit Amsterdam, Amsterdam 1081BT, The Netherlands; ^2^ Department of Organization Sciences, Vrije Universiteit Amsterdam, Amsterdam 1081HV, The Netherlands; ^3^ Department of Psychology, University of Koblenz-Landau, Landau, Germany; ^4^ Department of Psychology, University of Copenhagen, København 1353, Denmark; ^5^ Centre for Research in Experimental Economics and Political Decision Making (CREED), University of Amsterdam, 1001NJ Amsterdam, The Netherlands; ^6^ Institute for Advanced Study in Toulouse, Université Toulouse 1 Capitole, Toulouse 31015, France; ^7^ CAS Key Laboratory of Behavioral Science, Institute of Psychology, Chinese Academy of Sciences, Beijing 100101, People's Republic of China; ^8^ Department of Psychology, University of Chinese Academy of Sciences, Beijing 100049, People's Republic of China

**Keywords:** gossip, reputation, experience sampling, indirect reciprocity, partner selection, cooperation

## Abstract

Gossip—a sender communicating to a receiver about an absent third party—is hypothesized to impact reputation formation, partner selection, and cooperation. Laboratory experiments have found that people gossip about others' cooperativeness and that they use gossip to condition their cooperation. Here, we move beyond the laboratory and test several predictions from theories of indirect reciprocity and reputation-based partner selection about the content of everyday gossip and how people use it to update the reputation of others in their social network. In a Dutch community sample (*N* = 309), we sampled daily events in which people either sent or received gossip about a target over 10 days (*n*_gossip_ = 5284). Gossip senders frequently shared information about targets’ cooperativeness and did so in ways that minimize potential retaliation from targets. Receivers overwhelmingly believed gossip to be true and updated their evaluation of targets based on gossip. In turn, a positive shift in the evaluation of a target was associated with higher intentions to help them in future interactions, and with lower intentions to avoid them in the future. Thus, gossip is used in daily life to impact and update reputations in a way that enables partner selection and indirect reciprocity.

This article is part of the theme issue ‘The language of cooperation: reputation and honest signalling’.

## 1. Background

Indirect reciprocity and reputation-based partner selection can enable large-scale cooperation among genetically unrelated individuals even when they have a low probability of future interaction [1–8]. Indirect reciprocity occurs when individual A helps (harms) another individual B, then individual C learns about this behaviour and subsequently decides to help (harm) A when they meet. Partner selection occurs when C uses information about A's past (un)cooperative behaviour towards B to select (or avoid) A for social exchange, friendship, coalition formation, or as a sexual/romantic partner (for reviews, see [5,9]). Cooperation through these mechanisms is evident in non-human organisms [10–12]. However, information sharing through *gossip* is unique to humans [5,13].

Gossip—a *sender* communicating to a *receiver* about a *target* who is absent or unaware of the content (for reviews of gossip definitions, see [14,15])—is a key element in reputation-based cooperation. Gossip is ubiquitous, dominates human conversations [16], and is observed across societies [1,17,18]. Gossip can enhance the spread of reputational information, expanding the potential for indirect reciprocity and reputation-based partner selection to promote cooperation. Laboratory research found that people indeed gossip about others' cooperative or uncooperative behaviour in ways that affect the targets’ reputation, which, in turn, enables reputation-based cooperation [19–25]. Although theory and experimental research suggest that gossip is a pervasive feature of human societies and can enable large-scale cooperation, there is a lack of rigorous and systematic observations of the antecedents and consequences of gossip in daily life, perhaps owing to the challenges of studying gossip in its natural context.

The current study provides such insights by using experience sampling methods that ask people to report on recent events in which they sent or received gossip in their daily lives. Experience sampling provides advantages over earlier research on gossip in daily life that used either recall methods or daily diary surveys [26,27], because reporting on gossip closer to its occurrence can reduce biases that are present in retrospective reports. Moreover, compared to studies that have used eavesdropping in public to study gossip [28–30], experience sampling can be used to acquire rich data about the relationships between the sender, receiver, and target of gossip. Therefore, experience sampling can overcome some limitations of previous approaches to the study of gossip and allowed us to directly investigate: (i) what content is commonly communicated in gossip, (ii) the perceived veracity of gossip in daily life, (iii) the quality of the relationships between the sender, receiver and target of gossip, and (iv) whether and how receivers use everyday gossip to infer and update the reputation of the gossip targets (e.g. trustworthiness) which may enable indirect reciprocity and partner selection.

### (a) Content of gossip in daily life

Based on theories of indirect reciprocity and partner selection, gossip should convey cues about whether others are trustworthy [31,32], such as information about norm violations (i.e. violating shared expectations). Indeed, people gossip about others' trustworthiness and norm violations in controlled laboratory settings [33,34], but the extent to which this occurs in natural contexts is essentially unknown. Further, modelling work has mostly conceptualized reputation as unidimensional with a particular focus on trustworthiness [2], but people are thought to evaluate others on multiple dimensions, including trustworthiness, as well as warmth, competence and dominance [35,36]. If gossip is a mechanism that allows reputation formation, we would expect these dimensions of person perception to be communicated through gossip [37]. Gossip about norm violations might especially contain information about whether the target has been cooperative (i.e. is a friend or foe), which would be reflected by corresponding descriptions of the target along the dimensions of trustworthiness and warmth. Negative evaluations of the target's trustworthiness and warmth can indeed impose reputational costs on the norm violator [31,38]. Here, for the first time, to our knowledge, we randomly sample reports of gossip in everyday life to observe: (i) whether people gossip about norm violations, (ii) whether gossip conveys targets' trustworthiness, warmth, competence, and dominance, and (iii) how gossip about norm violations is associated with the portrayal of gossip targets.

### (b) Beliefs about the veracity of gossip

For gossip to facilitate indirect reciprocity and partner selection, it is essential that gossip is true [5,39–41] or that people can detect when gossip is false [40]. Because gossip can be easily manipulated, people may be motivated to share false or exaggerated gossip (i.e. to damage the reputation of a competitor, [42–45]). Therefore, humans may have evolved psychological adaptations that enable them to infer the veracity of gossip from cues [40,46]. Specifically, we examine: (i) whether receiving gossip from multiple independent sources is associated with increases in the perceived veracity of gossip [40], and (ii) whether detecting competing (versus corresponding) interests between senders and targets is associated with decreases in the perceived veracity of gossip [40].

### (c) Gossip that evades retaliation

Gossip can be used to indirectly punish or indirectly aggress against (non-cooperative) targets by imposing reputational costs on them [42,45,47–50]. Targets may, therefore, retaliate if they learn about gossip including negative content. To avoid the potential costs of retaliation, people may gossip in ways that minimize the chance of detection [17,47,51]. This implies that senders should mostly gossip to close, trustworthy others. More generally, senders may only gossip to receivers who are unlikely to expose them to the target [27,52,53], such as receivers that do not have a highly valued relationship with the target [54]. Thus, gossip instances that describe norm violations or other negative content should be more likely to occur in coalition structures characterized by: (i) a positive, highly valued relationship between the sender and the receiver, and (ii) a mutual negative, less valued relationship between the sender/receiver and the target [50,55,56].

### (d) The social consequences of gossip

According to theories of indirect reciprocity and partner selection, information shared through gossip is used to form and update a target's reputation [2,17,20,57]. Gossipers share information about others' (un)trustworthiness (e.g. about norm violations) that is essential in selecting cooperative partners and avoiding free-riders [16,33,58], thus ensuring future cooperation. Therefore, we expect that receiving gossip about a target's trustworthiness will (i) predict a change in the extent to which receivers value their relationship with targets, which in turn will (ii) be associated with their intentions to help, confront, and avoid targets.

## 2. Methods

### (a) Participants

We recruited 309 Dutch participants (32.4% male, 67.6% female; *M*_age_ = 39.51 years, s.d. = 16.92, range 18–75 years; *median* monthly income = €1600–€1999; vocational training (38.5%) was the most common education, followed by completed university (32.0%); 90.2% were born in The Netherlands, with 26.5% having at least one parent born outside The Netherlands). Participants received €20 for the intake session, €0.50 per completed experience sampling survey (maximum €20) and a €20 bonus for completing at least 80% of the experience sampling surveys (*M*_earnings_ = €51.62, s.d. = 12.29). Data were collected from 9 April 2018 to 28 June 2018.

### (b) Materials and design

Participants first completed a signup, including information about the study and inclusion criteria (fluent in Dutch, age greater than or equal to 18 years, owning a smartphone with internet access).

#### (i) Intake session

Participants first provided informed consent. Then, participants indicated the initials of 15 people from their social network that *they most frequently interacted with* in daily life. For each person, participants rated closeness (‘I feel close to [initials].’, 1 = *not at all close*, 7 = *extremely close* [59]), conflict (‘What is good for [initials] is good for me.’ [60]), welfare trade-off ratio (WTR) (i.e. give up an amount from €0 to €10, for [initials] to earn €10, ‘My relationship with [initials] is very important to me’; adapted from [61]) and trust (‘I trust [initials].’, ‘[initials] is concerned for my welfare’ adapted from [62]). The items for conflict (reverse scored) and trust were rated from 1 = *completely disagree* to 7 = *completely agree*. We aggregated measures of closeness, conflict, WTR and trust into a single index of relationship value (Cronbach's *α* = 0.89; see the electronic supplementary material).

Finally, participants received detailed instructions about the experience sampling survey, including the survey questions and the events they would report. To prevent negative associations, we did not explicitly mention the term gossip. The instructions and items in the experience sampling survey were selected through an extensive pilot study (see the electronic supplementary material and https://osf.io/xkn2z/files/).

#### (ii) Experience sampling phase

The experience sampling phase began the day after the intake session. For 10 consecutive days, participants received four text messages each day through SurveySignal [63] at a random time in each of four timeslots (i.e. 10.45–11.15, 13.45–14.15, 16.45–17.15 and 19.45–20.15). Participants received a survey link via text messages. Survey links remained open for 60 min, after which the instance was coded as missing. If participants did not open the link, a reminder was sent after 15 min.

Participants were asked whether they had experienced a situation, since receiving the last message, in which *they sent (or received) information to (from) another person about another person who was absent or had no knowledge of the communicated information*. If so, they were asked to report the last such situation they experienced. Participants were randomly assigned to report about either sending or receiving gossip*.* If one of the situations did not occur (e.g. sending/receiving gossip) they were asked about the other situation (e.g. receiving/sending gossip). If participants experienced neither sending nor receiving gossip, they were asked to report on other situations irrelevant to the current manuscript (e.g. what they were doing at that moment). Participants could not skip reporting (other than closing the survey) and were always asked to complete only one situation report (e.g. either sending or receiving gossip but not both; see the electronic supplementary material). Overall, we obtained 9923 responses (response rate: 80.1%; median response rate per participant: 87.5%; sending gossip: *n* = 2516; receiving gossip: *n* = 2768).

Participants indicated whether the receiver (or sender) and the target of gossip were part of their reported network. If so, participants could select their initials. If not, participants indicated the gender, social network layer (see the electronic supplementary material) and type of relationship. Participants described the gossip in one to three sentences (see the electronic supplementary material for examples of gossip descriptions).

Next, participants completed measures about the content of gossip and the involved parties, including gossip valence (‘How positive or negative was the information you communicated/received about the target?’ 1 = *extremely negative*, 4 = *neutral,* 7 = *extremely positive*) and whether gossip was relevant to a norm violation (‘Was the gossip about the target violating a social norm or rule?’ 0 = *no*, 1 = *yes*). For additional measures, see the electronic supplementary material.

Participants indicated how the information portrayed the target along four dimensions (trustworthiness, warmth, competence, and dominance; [27,29,30]). The items were ‘based on the information, how (i) trustworthy/honest (1 = *untrustworthy/dishonest*, 7 = *trustworthy/honest*), (ii) warm/agreeable (1 = *cold/disagreeable*, 7 = *warm/agreeable*), (iii) competent/knowledgeable (1 = *ignorant/incompetent*, 7 = *knowledgeable/competent*), and (iv) powerful/dominant (1 = *weak/submissive*, 7 = *powerful/dominant*) was the target?’ In the 7-point scales, 4 indicated *neutral* and 0 indicated irrelevant (see the electronic supplementary material).

Participants rated the number of times they previously received the information (0 = *not received previously,* 1 = *one time* … 10 = *10 times*, 11 = *more than 10 times*), the number of sources that shared the information (0 = *only the reported source,* 1 = *one additional source* … 10 = *10 additional sources*, 11 = *more than 10 additional sources*) and the overall perceived veracity of the information (‘To what extent do you believe the information is true?'; 1 = *definitely false*, 7 = *definitely true*).

Participants evaluated their own relationship with the receiver (or sender) and the target, as well as the relationship between the receiver (or sender) and the target using the same measures for closeness, trust and WTR as at intake, but each item was preceded by ‘At the moment…’. Different from other measures, we measured the relationship between the receiver/sender and the target using only one item for trust (the sender/receiver trusts the target) and did not include the items for WTR. Again, items were collapsed into a single relationship value index (ranging from 0 to 100; *α*s > 0.85; see the electronic supplementary material).

Lastly, participants reported their intentions to help, avoid, and confront the target (‘I would … go out of my way to help/avoid/confront the target’; 1 = *completely disagree*, 7 = *completely agree*).

### (c) Statistical analyses

The sample size varies between analyses because some participants dropped out of the experience sampling phase after providing only a few responses (and we did not exclude incomplete responses from the analyses). For analyses using continuous outcome variables, mixed-effects models with random intercepts for participants were conducted in R [64] using the packages ‘lme4’ [65], ‘lmerTest’ [66], ‘r2glmm’ [67], ‘emmeans’ [68], and ‘mediation’ [69] for mediation analyses. When sending and receiving gossip were combined in a model, we controlled for sending versus receiving gossip. Proportions were compared with an equality of proportions test without continuity correction. For analyses with binary outcome variables, we used generalized estimating equations in SPSS. All reported coefficients are unstandardized. Our analyses did not control for gender, but we additionally report tests of gender differences in each dependent variable (see the electronic supplementary material).

## 3. Results

We documented a large and diverse sample of gossip in daily life (*n* = 5284). Gossip varied in the medium of communication (68.4% face-to-face), the source of information (74.9% through first-hand experience), the number of people involved (73.7% dyads), and formal versus informal settings (82.8% informal; see the electronic supplementary material).

### (a) Content of gossip

Reports of sent and received gossip varied across the entire scale of valence. We validated the self-reported valence ratings using automatic text analysis of sentiment valence. We found small to moderate positive correlations between the valence rated by text analysis and participants' self-report ratings of the gossip's valence (*r*s > 0.20, *p*s < 0.001; see the electronic supplementary material).

Overall, 31.5% of gossip messages were reported as neutral (middle anchor), while 68.5% contained some evaluative content about the target (35.9% negative (below middle anchor) and 32.6% positive (above middle anchor)). Moreover, using the classification above (*n*_gossip_ = 5242), participants reported gossip that portrayed the target as negative slightly more frequently compared to neutral (*b* = 0.20, odds ratio = 1.21, Wald $\chi_{1}^{2}=14.85$, *p* < 0.001) or positive (*b* = 0.14, odds ratio = 1.15, Wald $\chi_{1}^{2}=7.95$, *p* = 0.005), respectively.

Across all reports, participants frequently reported gossip as being relevant to evaluating the target's trustworthiness (60.6%), warmth (61.0%), competence (59.2%) and dominance (61.1%), with no significant difference in the frequency of these dimensions, $\chi_{3}^{2}=5.11$, *p* = 0.164. Thus, gossip enabled people to frequently share information relevant to evaluating other's reputations on four key dimensions of person perception.

**Figure 1.**
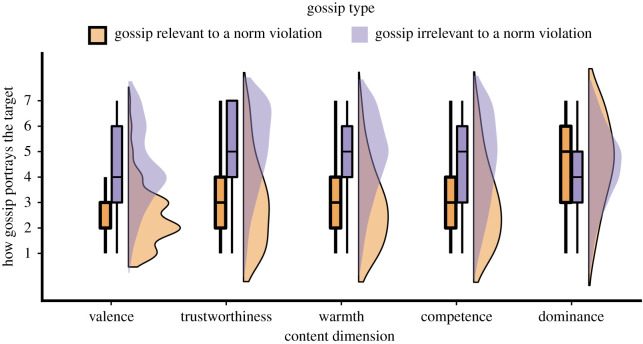
The content of gossip in daily life. Note. Distribution of the ratings of gossip content, comparing gossip relevant to a norm violation and with gossip irrelevant to a norm violation, for valence (1 = *extremely negative*, 7 = *extremely positive*); trustworthiness (1 = *untrustworthy/dishonest*, 7 = *trustworthy/honest*), warmth (1 = *cold/disagreeable*, 7 = *warm/agreeable*); competence (1 = *ignorant/incompetent*, 7 = *knowledgeable/competent*); and dominance (1 = *weak/submissive*, 7 = *powerful/dominant*); in all dimensions 4 = *neutral*. The box limits show the 25th percentile, the median and the 75th percentile, respectively. The whiskers extend to 1.5 times the interquartile range [70].

People reported gossip about the target violating a social norm/rule in 14.8% of all reports. Gossip relevant to norm violations, compared to gossip irrelevant to norm violations, portrayed targets largely more negatively (*n* = 5242, *b* = −1.82, *t*_5,235.79_ = −31.27, *p* < 0.001, semi-partial *r^2^* = 0.16), as well as largely less trustworthy (*n* = 3175; *b* = −1.99, *t*_3,148.92_ = −28.25, *p* < 0.001, semi-partial *r^2^* = 0.19), largely less warm (*n* = 3198; *b* = −1.88, *t*_3,188.55_ = −26.63, *p* < 0.001, semi-partial *r^2^* = 0.18), and largely less competent (*n* = 3101; *b* = −1.52, *t*_3,073.69_ = −19.51, *p* < 0.001, semi-partial *r^2^* *=* *0*.10), but slightly more dominant (*n* = 3199; *b* = 0.15, *t*_3195_ = 2.22, *p* = 0.026, semi-partial *r*^2^ = 0.002; see figure 1).

### (b) Beliefs about the veracity of gossip

Overall, participants overwhelmingly reported believing gossip to be true (*M* = 6.37, s.d. = 1.01; 63.0% reported received gossip as definitely true). Moreover, they perceived gossip as slightly less true when reporting the sender and target to have a conflict of interests (*n* = 2736; *b* = −0.05, *t*_2715_ = −3.95, *p* < 0.001, semi-partial *r*^2^ = 0.01), and when reporting a conflict of interests between themselves and the sender (*b* = −0.11, *t*_2593_ = −7.26, *p* < 0.001, semi-partial *r*^2^ = 0.02). However, participants' own conflict with the target, the number of sources, and the number of times people received the same gossip did not predict perceived gossip veracity (*p*s > 0.168, semi-partial *r^2^* ≤ 0.001).

### (c) Gossip that evades retaliation

Participants assigned a largely higher relationship value to their gossip partner (*M* = 71.30, s.e. = 0.68) than to the target (*M* = 47.50, s.e. = 0.68; *n* = 15 681; *b* = −23.79, *t*_15 354.18_ = −53.02, *p* < 0.001, semi-partial *r*^2^ = 0.13), and the relationship value participants assigned to their gossip partner was slightly higher than between the partner and the target (*M* = 63.50, s.e. = 0.68; *b* = −7.77, *t*_15 354.23_ = −17.32, *p* < 0.001, semi-partial *r*^2^ = 0.02).

**Figure 2.**
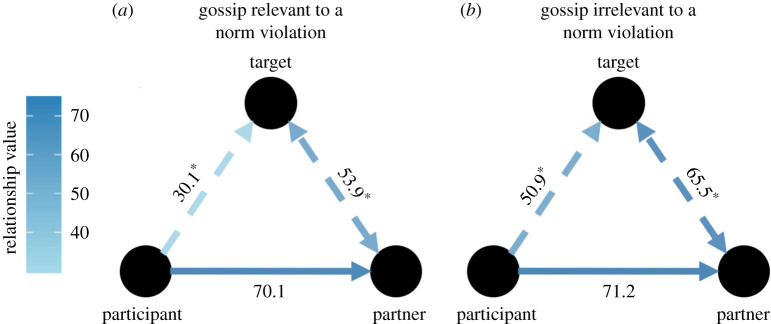
Relationship between the parties involved in gossip. Note. The three lines with arrows depict the relationship value between the participant and the gossip partner (sender and receiver combined), the participant and the gossip target, and the participant's perception of the relationship value between the partner and target for (*a*) gossip relevant to a norm violation and (*b*) gossip irrelevant to a norm violation. Darker colours represent higher relationship values. *significant difference (*p* < 0.001) between the corresponding values for gossip relevant to a norm violation and gossip irrelevant to a norm violation.

There was a significant interaction between (i) whether gossip was about a norm violation (or not), and (ii) the type of relationship in the gossip triad (i.e. participant–partner (sender/receiver), participant–target, partner (sender/receiver)–target) predicting relationship value (*F*_2,15 352_ = 119.19, *p* < 0.001, $\eta_{\mathrm{partial}}^{2}=0.02$). As figure 2 shows, compared to gossip that was irrelevant to norm violations, when people reported gossip about a norm violation, they assigned (i) moderately less value to their relationship with the target (*n* = 5224, *b* = −20.81, *t*_5204.04_) = −19.75, *p* < 0.001, semi-partial *r*^2^ = 0.07), and (ii) slightly less value to the relationship between the receiver and the target (*n* = 5228, *b* = −11.64, *t*_5215.82_ = −12.88, *p* < 0.001, semi-partial *r*^2^ = 0.03). We found this same pattern of interaction when comparing positive to negative gossip (see the electronic supplementary material). Taken together, these findings support the notion of a coalitional structure underlying gossip about norm violations (and negative gossip): the sender and receiver share a positive relationship, but they each have a less positive (and even negative) relationship with the target (figure 2).

### (d) Social consequences of gossip

**Figure 3.**
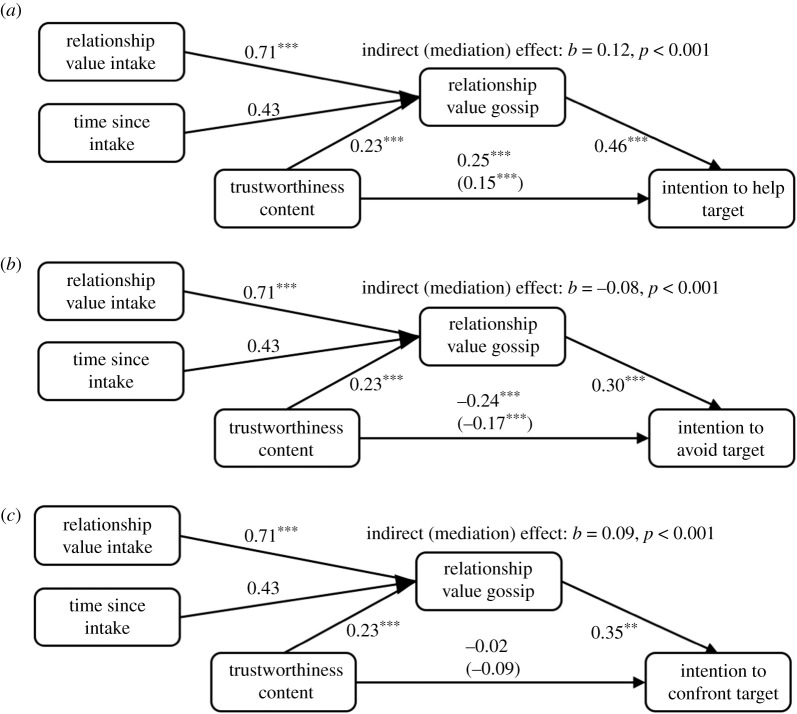
Change in relationship value assigned to the target mediates the effect of gossip content on behavioural intentions. Note. Three mediation models show the indirect effect of the gossip content describing the target's trustworthiness on intentions to (*a*) help, (*b*) avoid, and (*c*) confront the target through a change in relationship value assigned to the target from intake to experience. We also controlled for the interaction between time since intake and relationship value at intake, which was not significant (see the electronic supplementary material). **p < 0.01; ***p < 0.001.

We focus on gossip received about targets who are members of participants’ reported network and which was relevant to evaluating the target's trustworthiness (*n* = 335).^1^ Figure 3 shows first that trustworthiness content had: (i) a large positive association with participants' intentions to help targets, (ii) a moderate negative association with their intentions to avoid targets, and (iii) a non-significant relationship with their intentions to confront targets. Second, the portrayal of targets as trustworthy had a large positive association with the relationship value assigned to the target controlling for intake relationship value (i.e. residualized change in relationship value), time since intake and the interaction between these two factors. Third, the relationship value assigned to the target had a large positive association with intentions to help and a small positive association with intentions to confront targets, but a moderate negative association with intentions to avoid targets. Finally, we found significant indirect effects of trustworthiness content through (residualized) change in relationship value on intentions to help, confront, and avoid targets (*b* = 0.12, 95% CI (0.08, 0.17); *b* = 0.09, 95% CI (0.04, 0.14); and *b* = −0.08, 95% CI (−0.12, −0.05), respectively). A similar pattern of results was found when considering other aspects of gossip content as predictors (e.g. valence, warmth, competence, dominance, and norm violations, see the electronic supplementary material). Thus, gossip that portrayed the target as more trustworthy increased the relationship value assigned to the target, and this updated relationship value was associated with greater intentions to help and confront targets, as well as with lower intentions to avoid targets (figure 3).

### (e) Auxiliary analyses: gender and gossip

Past theory and research have discussed possible gender differences in the use of gossip as a form of indirect aggression [45,71,72]. We ran additional analyses that included gender predicting the outcomes of the analyses we report above. Overall, we found little to no gender differences in: (i) the content of gossip, (ii) the perceived veracity of gossip, and (iii) the relationship context of gossip (see the electronic supplementary material). However, when analysing the content of the gossip participants received, we found that when gossip was sent by women compared to men, then gossip was slightly: (i) more (frequently) negative, (ii) less often neutral, and (iii) more often about norms violations. The reported gender of the sender, however, was not associated with the perceived veracity of gossip or the relationship context of gossip. Men, compared to women, self-reported a slightly greater willingness to help and confront targets of gossip.

## 4. Discussion

According to theoretical models of the evolution of large-scale cooperation, gossip enables and increases cooperation in social networks. Laboratory experiments have indeed demonstrated that gossip can fulfil these roles [2,3,19,20,24,25]. Here, we had a community sample report on up to 40 events in which they either sent or received gossip over a 10 day period. Most gossip in daily life involved information that was acquired through first-hand experience (75%), was communicated face-to-face (68%) and occurred within dyads (74%). Supporting theories of indirect reciprocity and partner choice, we found that: (i) people do gossip about information that can be used to evaluate others’ cooperativeness (e.g. trustworthiness, warmth, and norm violations), (ii) people use cues (e.g. conflict between the sender and target of gossip) to infer when gossip may be false, (iii) gossip most frequently occurs in ‘coalition network’ structures, in which the sender and receiver of gossip share a positive relationship and both have a less positive (even negative) relationship with the target [55,56], (iv) gossip is associated with (changes in) targets' cooperative reputation, and (v) this updated reputation can explain how the trustworthiness of the target as portrayed by the gossip predicts behavioural intentions toward the target of gossip in future interactions. This evidence demonstrates that gossip in daily life can have a role in facilitating cooperation, such as by enabling cooperators to assort and imposing costs on non-cooperators.

To capture multiple aspects of reputation, we asked people to what extent gossip portrayed the target according to key dimensions of person perception, including trustworthiness, warmth, competence, and dominance [36], which all relate to individuals’ cooperative reputation (see the electronic supplementary material). We found that gossip frequently contained content that could indeed be used to evaluate these characteristics. However, the descriptions of the target's trustworthiness, warmth, and competence in the gossip content were strongly intercorrelated. By contrast, dominance had a small, positive association with trustworthiness and warmth, but a strong positive association with competence (see the electronic supplementary material). This pattern of correlations between the different characteristics in how gossip portrayed targets is aligned with the valence-dominance framework [73], but may also reflect the method of measurement (i.e. single item measures in experience sampling), a halo-effect [74], or a might-over-morality effect [75]. Future work will need to further examine the multi-dimensionality of partner qualities that can be inferred from gossip. Nonetheless, the current findings suggest that future research should consider how these different dimensions of person perception can be integrated into models of reputation-based cooperation (also see [76] for different domains of reputation). Future theoretical models can include agents that vary on these attributes, consider how different behaviours impact these evaluations in gossip, and model strategies that use evaluations of these different dimensions to condition cooperation.

Gossip can serve as a means to indirectly punish norm violators [25,41–43,47–49,77] by negatively affecting their reputation, reducing their social standing in a group, lowering their chance to be selected as social exchange partners, and promoting social exclusion [20,45]. Relative to directly confronting and punishing norm violators, gossip can circumvent the potential cost of retaliation, so long as the identity of the gossiper remains unknown to the target. In line with this reasoning, people were more likely to share gossip in ways that reduced the potential of detection and subsequent retaliation, such as gossiping to others who were described as being moderately more valued, close, and trusted, and when both sender and receiver had a slightly lower valued relationship with the target of gossip. We found some evidence that this strategy was particularly used when sharing more negative gossip (e.g. about a norm violation) that could lower the target's reputation [50,56]. Another strategy to evade retaliation is to obscure the source of information, such as verbally indicating they heard it from another person [51,78], which could be further investigated in daily life settings involving different gossip content and relationship contexts.

However, gossip may also be used to establish and maintain social bonds [41,42]. Consistent with this, we found that sharing more positive gossip with a person from one's social network was associated with a slight increase in relationship valuation (see the electronic supplementary material). Thus, besides imposing reputational costs on non-cooperators, gossip can serve other functions including social bonding between senders and receivers. Future research can further examine different functions of gossip and how these relate to strategies of gossip in different relationship contexts.

In order for gossip to facilitate indirect reciprocity to support cooperation, gossip must have a relatively high degree of accuracy [41,42], and one challenge to indirect reciprocity is that people can manipulate and spread false gossip [40,41,44]. Our current data do not allow inferences about whether the gossip was truthful or not, but we do know that people overwhelmingly *believed* the gossip they received. Correspondingly, laboratory research shows that people generally perceive gossip as true and act on it accordingly [79,80]. We did find support for the hypothesis that people were (slightly) less likely to believe gossip when there was a conflict of interests between senders and targets of gossip [40]. However, we found no support for the hypothesis that people are more likely to believe gossip when hearing the same gossip more frequently, either from the same or different senders. Future research could further investigate whether gossip is largely true or whether people are unable to infer gossip veracity by comparing gossip to known facts (cf. [72,81]). In the current study, we documented self-reported veracity assessments, and it is possible that participants were motivated to believe gossip because it supported their beliefs [79,82]. This could be investigated by relating a desire for information to be true with the perceived veracity of gossip, such as preferring negative (positive) gossip when it concerns rivals (allies; [44]).

The current study has some limitations that must be considered. First, our observational data of gossip in daily life does not allow causal inferences. However, our use of the network at intake allows us to make claims about changes in how people evaluate their relationship with people in their network after receiving gossip. Second, we only measured behavioural intentions, which hinders drawing conclusions about how gossip impacts actual cooperative behaviour towards targets. It is possible that there is a gap between intentions and behaviour [83]. Finally, as participants reported to the researchers, they may have refrained from reporting certain kinds of gossip, such as negative or false gossip. Nevertheless, we did not use the term gossip throughout the study, which should minimize biased reporting.

To conclude, theories of indirect reciprocity and partner selection focus on gossip that is related to the target's cooperativeness (i.e. trustworthiness), and gossip about a target's trustworthiness may influence individuals' evaluations of targets and their behavioural intentions toward targets [2,3,16,20,84]. We indeed found that people gossip about others’ trustworthiness and that this aspect of gossip was strongly positively associated with the relationship value that people assigned to targets of gossip, after controlling for relationship value at an earlier time point. Furthermore, these variations in relationship value had small to large associations with intentions to help, avoid, and confront gossip targets. Thus, people were more inclined to benefit a person who was described as trustworthy through gossip, and were less inclined to exclude them in social interactions. By showing how a single gossip statement can have consequences for a person's reputation and others' behavioural intentions towards them in future interactions, these findings illustrate how gossip in daily life can fuel a system of indirect reciprocity and partner selection that can informally regulate large-scale cooperation.

## Endnote

1 Because there were not enough cases with more than one report to reliably estimate effects within the experience sampling data, we analysed only the first gossip instance about a specific person from the network.

## Data accessibility

The materials, data, and code used in this study are openly accessible on the Open Science Framework: https://osf.io/xkn2z/files/.
